# The hydrogenosomes of *Psalteriomonas lanterna*

**DOI:** 10.1186/1471-2148-9-287

**Published:** 2009-12-09

**Authors:** Rob M de Graaf, Isabel Duarte, Theo A van Alen, Jan WP Kuiper, Klaas Schotanus, Jörg Rosenberg, Martijn A Huynen, Johannes HP Hackstein

**Affiliations:** 1 Department of Evolutionary Microbiology, IWWR, Radboud University Nijmegen, Heyendaalseweg 135, 6525AJ Nijmegen, The Netherlands; 2 Center for Molecular and Biomolecular Informatics, Nijmegen Center for Molecular Life Sciences, Radboud University Nijmegen Medical Centre, Geert Grooteplein 28, 6525GA Nijmegen, The Netherlands; 3 Sommerhaus 45, D-50129 Bergheim, Germany; 4 CIHR Group in Matrix Dynamics, University of Toronto, 150 College Street, Toronto, Ontario, Canada M5S 3E2; 5 Department of Ecology and Evolutionary Biology, 106A Guyot Hall, Princeton University, Princeton NJ 08554-2016, USA; 6 Netherlands Bioinformatic Centre, Geert Grooteplein 28, 6525 GA Nijmegen, The Netherlands

## Abstract

**Background:**

Hydrogenosomes are organelles that produce molecular hydrogen and ATP. The broad phylogenetic distribution of their hosts suggests that the hydrogenosomes of these organisms evolved several times independently from the mitochondria of aerobic progenitors. Morphology and 18S rRNA phylogeny suggest that the microaerophilic amoeboflagellate *Psalteriomonas lanterna*, which possesses hydrogenosomes and elusive "modified mitochondria", belongs to the Heterolobosea, a taxon that consists predominantly of aerobic, mitochondriate organisms. This taxon is rather unrelated to taxa with hitherto studied hydrogenosomes.

**Results:**

Electron microscopy of *P. lanterna *flagellates reveals a large globule in the centre of the cell that is build up from stacks of some 20 individual hydrogenosomes. The individual hydrogenosomes are surrounded by a double membrane that encloses a homogeneous, dark staining matrix lacking cristae. The "modified mitochondria" are found in the cytoplasm of the cell and are surrounded by 1-2 cisterns of rough endoplasmatic reticulum, just as the mitochondria of certain related aerobic Heterolobosea. The ultrastructure of the "modified mitochondria" and hydrogenosomes is very similar, and they have the same size distribution as the hydrogenosomes that form the central stack.

The phylogenetic analysis of selected EST sequences (Hsp60, Propionyl-CoA carboxylase) supports the phylogenetic position of *P. lanterna *close to aerobic Heterolobosea (*Naegleria gruberi*). Moreover, this analysis also confirms the identity of several mitochondrial or hydrogenosomal key-genes encoding proteins such as a Hsp60, a pyruvate:ferredoxin oxidoreductase, a putative ADP/ATP carrier, a mitochondrial complex I subunit (51 KDa), and a [FeFe] hydrogenase.

**Conclusion:**

Comparison of the ultrastructure of the "modified mitochondria" and hydrogenosomes strongly suggests that both organelles are just two morphs of the same organelle. The EST studies suggest that the hydrogenosomes of *P. lanterna *are physiologically similar to the hydrogenosomes of *Trichomonas vaginalis *and *Trimastix pyriformis*. Phylogenetic analysis of the ESTs confirms the relationship of *P. lanterna *with its aerobic relative, the heterolobosean amoeboflagellate *Naegleria gruberi*, corroborating the evolution of hydrogenosomes from a common, mitochondriate ancestor.

## Background

Aerobic eukaryotes possess classical mitochondria, but anaerobic eukaryotes can host very diverse organelles that belong to a broad spectrum of double-membrane bounded, mitochondria-related compartments. These organelles range from full-fledged, but anaerobic mitochondria to tiny "mitosomes" with a minimal protein content. Examples of these anaerobic functioning organelles are the "mitochondria-like" organelles of *Blastocystis *[1], the "mitochondrial remnant" of *Cryptosporidium *[2], the "hydrogenosomes" of *Trichomonas *[3] and the "mitosomes" of *Giardia, Entamoeba*, and *Trachipleistophora *[4-6]. Anaerobic mitochondria, mitochondria-like organelles and hydrogenosomes produce ATP, albeit with either different electron transport chains than in aerobic mitochondria, or without an electron transport chain altogether. Mitosomes do not produce ATP - they seem to host only enzymes engaged in Fe-S cluster biogenesis [7]. The presence of these proteins appears to be the only property that is shared between all members of the mitochondrial family, perhaps with the exception of the mitosomes of *Entamoeba histolytica *and *Mastigamoeba balamuthi *where the corresponding proteins are likely to be localized in the cytoplasm [8-10]. Hydrogenosomes produce molecular hydrogen with the aid of one or several hydrogenases. They are double-membrane bounded organelles sized approximately 0.5 - 2 μm. They are found in a broad spectrum of unicellular, anaerobic (or microaerophilic) protists such as parabasalid flagellates (*Trichomonas vaginalis, Tritrichomonas foetus, Histomonas meleagridis*), excavate, preaxostylid flagellates (*Trimastix pyriformis*), heterolobosean amoeboflagellates (*Psalteriomonas lanterna*), anaerobic ciliates (e.g. *Nyctotherus ovalis, Metopus palaeformis, Trimyema compressum, Caenomorpha uniserialis, Dasytricha ruminantium*), and anaerobic chytridiomycete fungi (*Neocallimastix sp., Piromyces sp*.). The broad phylogenetic distribution of their hosts suggests that the hydrogenosomes of these organisms evolved several times independently. Accordingly, hydrogenosomes are not the same, they differ structurally and metabolically [11-14]. However, it is likely that all these various hydrogenosomes produce ATP by substrate-level phosphorylation. Besides ATP and hydrogen, most of them produce CO_2 _and acetate as end products of their carbohydrate metabolism. *Nyctotherus ovalis *produces succinate in addition, and the ciliate *Trimyema compressum *as well as the anaerobic chytrids *Neocallimastix sp*. and *Piromyces sp*. produce formate as one of their metabolic end products [11,12,15,16]. The major substrate of the carbohydrate catabolism of hydrogenosomes is pyruvate that is metabolized by either pyruvate:ferredoxin oxidoreductase (PFO) as in *Trichomonas *or pyruvate:formate lyase (PFL) as in *Neocallimastix *and *Piromyces *[15,17]. Notably, *Nyctotherus ovalis *uses pyruvate dehydrogenase (PDH) as aerobic mitochondria do [16]. For their major redox-reactions, hydrogenosomes use ferredoxins or components of a mitochondrial or bacterial complex I. [18,19].

Evidence from morphology and 18S rRNA phylogeny suggests that the microaerophilic amoeboflagellate *Psalteriomonas lanterna *belongs to the Heterolobosea, a taxon that consists predominantly of aerobic, mitochondriate organisms [20-23]. Only three related anaerobic organisms, the amoebae *Vahlkampfia anaerobica, Monopylocystis visvesvarai *and *Sawyeria marylandensis *have been described. While the lack of molecular data does not allow a closer determination of the phylogenetic position of *Vahlkampfia anaerobica*, 18S rRNA data clearly reveal that the latter two amoebae are close relatives of *Psalteriomonas lanterna *[22-24]. The flagellate stage of *Psalteriomonas lanterna *hosts a large globular hydrogenosomal complex that is associated with numerous endosymbiotic methanogens [20]. Remarkably, it also hosts 0.6 - 3.0 μm sized cytoplasmic organelles that were interpreted as "modified mitochondria" [20,25]. If this interpretation is true, it would make *Psalteriomonas *unique in having both mitochondria and hydrogenosomes which are normally mutually exclusive. The large, globular organelles were identified as hydrogenosomes using a cytochemical reaction (BSTP staining, c.f. [26]) to detect hydrogenase activity and the organelle's reaction with a heterologous antibody against hydrogenase [25]. The "modified mitochondria" reacted only weakly with the antibody and were not analyzed in more detail. Physiological studies were not performed since *P. lanterna *cannot be cultured axenically. Molecular information is restricted to the DNA sequence of a ferredoxin and the 18S rRNA gene; the latter allowed the determination of the phylogenetic position of *P. lanterna *as belonging to the Percolozoa (Heterolobosea) with a sistergroup relationship to the Vahlkamphidae [21-23,27].

Here we present a combined electron microscopic and molecular study that aims to unravel the structure and function of the hydrogenosomes and the presumed "modified mitochondria" of *Psalteriomonas lanterna*. We describe the ultrastructure of the "modified mitochondria" and hydrogenosomes in detail and provide evidence that both organelles are actually two morphs of the same organelle and not two different organelles. Moreover, we provide molecular information from preliminary EST studies on the phylogenetic position with respect to the aerobic relatives and the potential function of the hydrogenosomes. These studies suggest that the hydrogenosomes of *P. lanterna *are physiologically similar to the hydrogenosomes of *Trichomonas vaginalis *and *Trimastix pyriformis *[17,28]. Phylogenetic analysis of the ESTs confirms the relationship of *P. lanterna *with its aerobic relative, the heterolobosean amoeboflagellate *Naegleria gruberi*. This organism is a free-living soil and freshwater amoeboflagellate and closely related to the pathogenic *Naegleria fowleri *that can cause severe amoebic meningitis.

## Results and discussion

### Electron microscopy

**Figure 1:**
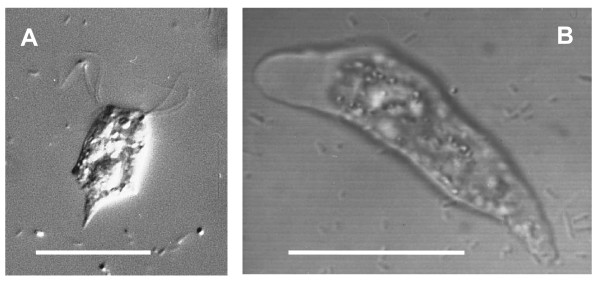
**Light microscopy of *Psalteriomonas lanterna***. A: Flagellate stage of *Psalteriomonas lanterna *DIC-microscopy. At the apical side of the cell two of the four flagella clusters can be seen. The globule in the centre of the cell is the hydrogenosomal complex. B: Amoeba stage of *Psalteriomonas lanterna*. CLS-microscope. Bars: 30 μm

Light microscopy of *Psalteriomonas lanterna *flagellates reveals a large globule in the centre of the cell (Fig. 1a). This globule had been identified as a hydrogenosome by its reaction with an antiserum against hydrogenase and activity staining for hydrogenase with the aid of the BSTP reaction [25]. Also in the amoeba stage this globule is present but less prominently shaped (Fig. 1b). DAPI - and ethidium bromide staining of the globule for nucleic acids were negative (not shown).

**Figure 2:**
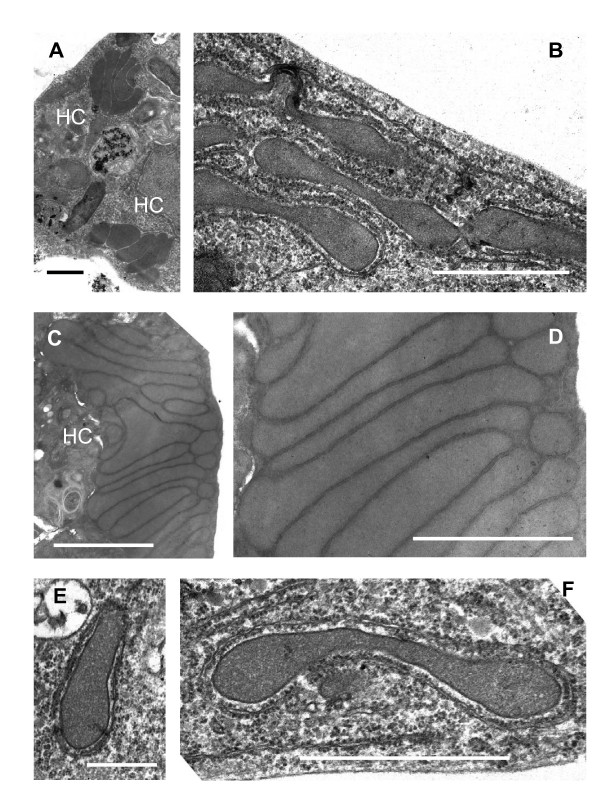
**Electron microscopy of the hydrogenosomes of *Psalteriomonas lanterna *flagellates**. A: Cell with two small stacks of hydrogenosomes. HC: hydrogenosomal complex. B: Group of dumb-bell-shaped hydrogenosomes in the periphery of the cell. The hydrogenosomes are surrounded by cisterns of rough endoplasmatic reticulum (rough ER). These organelles have been named "modified mitochondria" by Broers (1992) [25]. C: Large stack of hydrogenosomes (HC). D: Detail of the hydrogenosomal complex shown in C. E: "Single" hydrogenosome surrounded by rough ER. F: Dumb-bell-shaped hydrogenosome ("modified mitochondrion"). Bars A-D, F: 1 μm; E: 0,5 μm

When the hydrogenosomes of *Psalteriomonas lanterna *were described for the first time at the electron microscopy level, they were seen to form globules consisting of closely packed "microbodies" intermingled with symbiotic methanogenic archaea [20]. In some cases the hydrogenosomes (microbodies) were penetrated by methanogens [25]. Notably, the analysis of symbiont-free cells revealed that the hydrogenosomes also assembled into globules in the absence of methanogenic archaea. After more than 20 years of cultivation, all *Psalteriomonas lanterna *cells became free of methanogens as judged by the absence of methane production and the specific F_420 _autofluorescence [29] (data not shown). Electron microscopical analysis confirms the absence of methanogens and reveals that the central globule is a large complex built up from stacks of more than 20 individual hydrogenosomes, which are predominantly sausage- and dumb-bell-shaped (Fig. 2c, d). Individual hydrogenosomes are surrounded by a double membrane that encloses a homogeneous, dark staining matrix (Fig. 2d). In a few cells, up to four smaller hydrogenosomal complexes were found; the stacks consist of 5-6 individual hydrogenosomes (Fig. 2a). These stacks are regarded as juvenile complexes.

**Figure 3:**
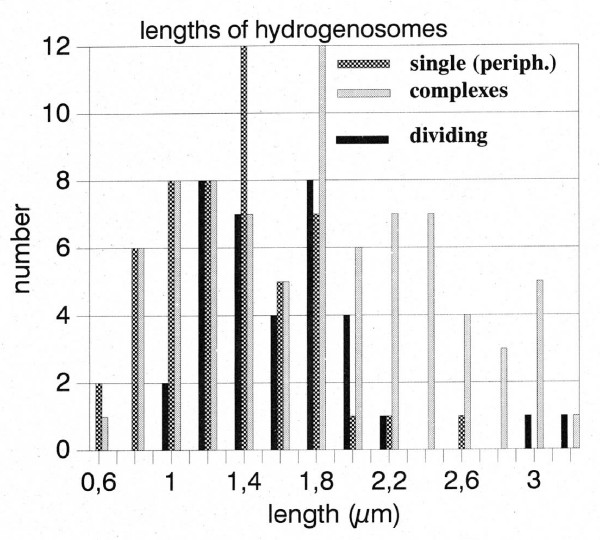
**Histogram of the lengths of hydrogenosomes**. Randomly selected sections of hydrogenosomes on the electron micrographs were measured and plotted. "dividing": dumb-bell-shaped organelles. "complexes": hydrogenosomes from stacks. All kinds of hydrogenosomes belong to the same length distribution.

Broers [20] described "modified mitochondria" in the periphery of the *Psalteriomonas lanterna *cells, odd organelles that were surrounded by a cistern of rough endoplasmatic reticulum (rough ER). In our study, these "modified mitochondria" look very similar to the individual hydrogenosomes of the hydrogenosomal complex of the globule (Fig. 2b, e, f). The matrix is homogeneous, but less densely stained as in the stacked hydrogenosomes; there is no evidence for the presence of (mitochondrial) cristae. All these organelles are surrounded by 1-2 cisterns of rough ER - like the mitochondria of aerobic Heterolobosea, e.g. *Tetramitus rostratus, Paratetramitus jugosus, Vahlkampfia aberdonica, Vahlkampfia avara *and *Vahlkampfia ustiana *[30,31]. Similarly to the hydrogenosomes in the stack, they are bounded by a double membrane. Most organelles are dumb-bell-shaped (Fig. 2b, f), some are sausage-shaped (Fig. 2e), and a few are cup-shaped and similar in appearance to the mitochondria of certain aerobic Heterolobosea, e.g. *Vahlkampfia ustiana *[31]. Certain dumb-bell- and cup-shaped organelles are rather slim in the middle, suggesting that these organelles might be fission stages similar to the fission stages of *Trichomonas vaginalis *hydrogenosomes [32]. A biometric analysis of the electron microscopic pictures of the stacked hydrogenosomes and the cytoplasmatic organelles revealed no differences in the length-distribution (Fig. 3). The diameter of the hydrogenosomes and the modified mitochondria show a modal distribution around 0.3 μm (range 0.1-0.9, N = 111; data not shown). Given the identical distribution of lengths and diameters and the very similar morphology, we conclude that the cytoplasmatic organelles are hydrogenosomes, potentially in young, dividing stages. Absence of staining of the cytoplasmatic organelles in the BSTP reaction at the light microscopic level can be explained either by a lack of hydrogenase activity in the "young" organelles or by an insufficient sensitivity of the BSTP reaction. On the other hand, pictures published by Broers [25] suggest a faint reaction with the hydrogenase antibody. Moreover, both types of organelles stain with Rhodamine-123 as normal mitochondria and hydrogenosomes [16,25]. Therefore, it is likely that both the stacked hydrogenosomes and the cytoplasmatic organelles ("modified mitochondria") are morphs (or developmental stages) of the same organelle. It is highly unlikely that hydrogenosomes and mitochondria occur in the same cell, because hydrogenosomes have been identified as a special kind of hydrogen-producing mitochondria [14,16,18]. On the other hand, different morphs of mitochondria have been described in the ciliate *Euplotes minuta *[33,34]. The most spectacular example of the presence of different mitochondria in the same organism is the "nebenkern" formation during spermatogenesis in *Drosophila *where mitochondria aggregate and fuse to form a globule of the same size as the nucleus [35].

### ESTs

About 480 randomly chosen clones were sequenced and analyzed using the BLAST X tool [36]. The clones were single reads of varying length. The genes discussed here (except 51 kDa) were extended by RT-PCR.

Although the cDNA library was created using poly-d(T) primers, several sequences of bacterial origin were identified that matched with species present in the non-axenic culture. However, bona-fide *Psalteriomonas *sequences were easily identified by their high A+T content (67-72%). In addition, a codon usage analysis was performed using the Cusp program from the EMBOSS package [37] and a principal component analysis. These analyses confirmed the homogeneity of the putative *Psalteriomonas *sequences.

The protein sequences were selected for phylogenetic analysis either because of their usefulness for establishing the phylogenetic position of *Psalteriomonas lanterna*: Elongation Factor 1 alpha, Hsp60, or for their potential role in the hydrogenosomal metabolism: putative ADP/ATP carrier, [FeFe] hydrogenase, pyruvate:ferredoxin oxidoreductase (PFO), propionyl-CoA carboxylase (PCCB), Complex I - 51 kDa subunit and glutamate dehydrogenase (GDH).

### Elongation Factor 1 alpha (EF-1 alpha)

During the translation of a mRNA chain in the ribosome, two GTPases play an important role in the elongation cycle: the Elongation factor 2 (EF-2 or EF-G in Prokaryotes) and the Elongation Factor 1 alpha. The EF-1 alpha (EF-Tu in Prokaryotes) is responsible for carrying and promoting the binding of aminoacyl-tRNAs to the A-site of the ribosomal small subunit [38].

**Figure 4:**
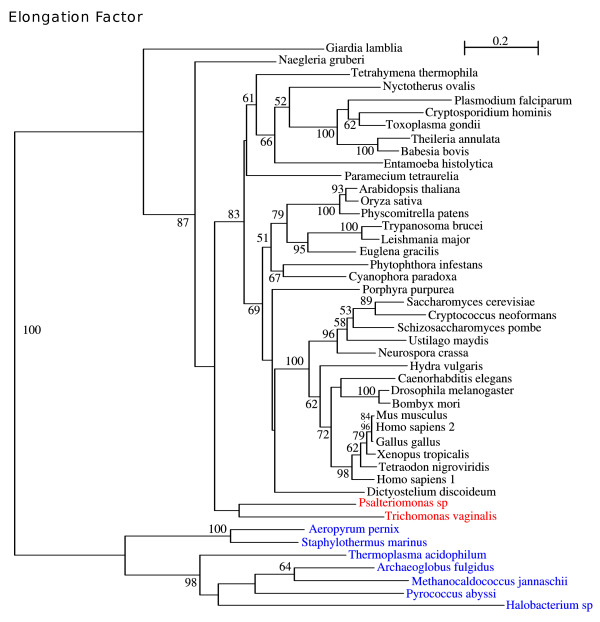
**ML tree for the Elongation Factor 1 alpha**. This tree was computed using the RtREV+4 discrete-rate G+I+F. The tree is rooted by an outgroup of Archaeal species (in blue). Branch values represent the bootstrap percentage.

Since Elongation Factor 1 alpha is present in the three domains of life, i.e., Bacteria, Archaea and Eukaryota, it should be a good phylogenetic marker that might be useful for inferring the phylogenetic position of *Psalteriomonas *within the eukaryotic tree of life. However, the species distribution within the tree calculated here is not in complete agreement with the eukaryotic tree of life since several species exhibit conspicuous artefactual relationships, particularly the polyphyly of the ciliates and Amoebozoa (Fig 4). Notwithstanding, *Psalteriomonas lanterna *clusters with *Trichomonas vaginalis *as seen in several of our phylogenies.

### Heat shock protein 60 (Hsp60/cpn 60)

Hsp60 (GroEL/cpn60) is an ATP-dependent, highly conserved protein, involved in protein folding, maturation, renaturation and assembly of complexes, as well as in intracellular cross-membrane shuttling of precursor-protein molecules [39]. In nearly all eukaryotes it is located in the mitochondria, hydrogenosomes or mitosomes.

Its structure resembles a cylindrical barrel, which binds and encloses the folding of proteins in its core [40]. It is an essential and highly conserved protein present in virtually all organisms, shows no evidence of horizontal gene transfer, and is frequently used as a mitochondrial marker [41].

**Figure 5:**
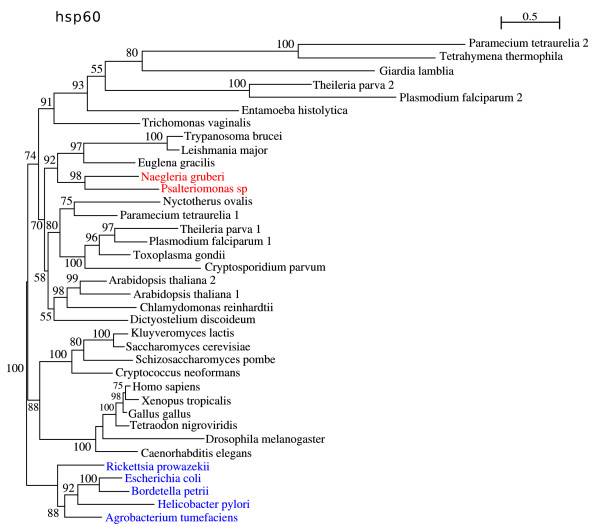
**Phylogeny of the Heat Shock Protein 60**. The branch values represent bootstrap values. ML tree computed with RtREV+4 discrete-rate G+I+F. An outgroup of Bacteria was chosen to root this tree (in blue).

The Hsp60 phylogeny shows the expected eukaryotic branching, and a solid bootstrap supported outgroup of prokaryotic sequences (Fig. 5). *Psalteriomonas lanterna *and *Naegleria gruberi *branch together corroborating a close relationship between the mitochondrion of *Naegleria gruberi *and the hydrogenosomes of *Psalteriomonas lanterna*. This clustering with *N. gruberi *is consistent with the previously published 18S rRNA phylogeny which groups both organisms [21-23]. Furthermore, the clustering of the Heterolobosea with the Euglenozoa seen in this tree is consistent with previously published eukaryotic phylogenies [42].

### Mitochondrial Solute Carrier (putative ADP/ATP Carrier (AAC))

The ADP/ATP translocator is a member of the Mitochondrial Carrier Family (MCF), which catalyses the transmembrane exchange of ATP produced in the mitochondria (or hydrogenosomes) for cytosolic ADP. All members of this protein family exhibit a tripartite structure which consists of three consecutive sequence repeats of about 100 residues each representing the 3 transmembrane domains [43]. This protein family is exclusively present in Eukaryotes.

**Figure 6:**
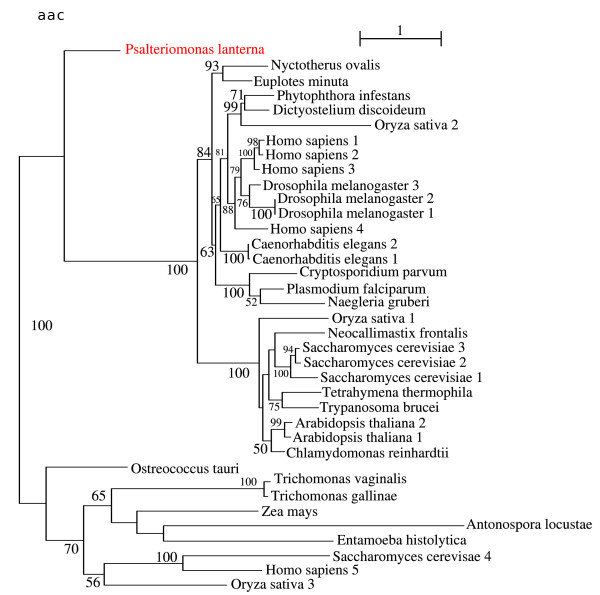
**ML phylogeny of the putative ADP/ATP carrier (member of the Mitochondrial Solute Carrier family)**. Branch values are the bootstrap percentages, and the tree was computed using a RtREV+4 discrete-rate G+F.

All bona-fide mitochondria, but also the hydrogenosomes of *Nyctotherus ovalis *and *Neocallimastix/Piromyces*, possess members of the mitochondrial-type ADP/ATP translocator subfamily. The hydrogenosomes of *Trichomonas vaginalis *and the mitosomes of *Entamoeba histolytica *and *Antonospora locustae *do not possess a mitochondrial-type ADP/ATP translocator. Instead they evolved alternative ADP/ATP carriers, which, of course, belong to the mitochondrial carrier family [44]. In addition, the three members of the mitochondrial carrier family of *Trimastix pyriformis *do not belong to the cluster of genuine mitochondrial-type AACs; their function has not yet been established [28]. While the AAC of *Naegleria gruberi *clusters within the bona-fide mitochondrial carriers, the mitochondrial carrier protein of *Psalteriomonas lanterna *assumes an intermediate position between the genuine mitochondrial AACs and the alternative transporters of *Trichomonas, Entamoeba *and *Antonospora *(Fig. 6). The phylogenetic position of the mitochondrial carrier protein of *Psalteriomonas lanterna *argues that this mitochondrial carrier might also be an alternative ADP/ATP carrier. Nevertheless, the alternative possibility, that the mitochondrial carrier protein of *Psalteriomonas lanterna *is derived from bona-fide AACs cannot be excluded.

### [FeFe] Hydrogenase

Hydrogenases are metalloenzymes that catalyze the reversible reaction that produces dihydrogen using two electrons and two protons. These enzymes are classified in three distinct classes according to the metallic composition of their prosthetic groups: the [Fe]-hydrogenases, only present in methanogenic Archaea; the [NiFe]-hydrogenases, widespread within prokaryotic organisms; and finally, the oxygen sensitive [FeFe]-hydrogenases [45].

**Figure 7:**
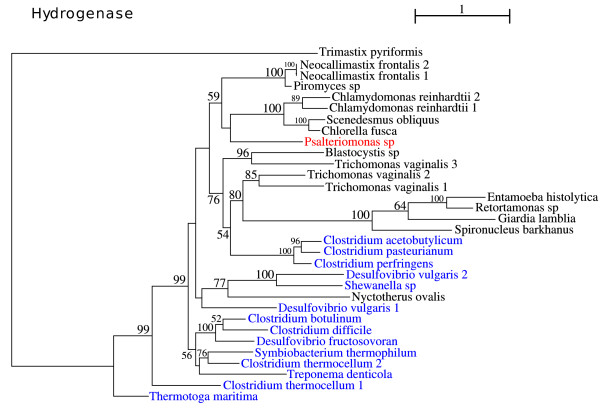
**Phylogeny of the [FeFe]Hydrogenase, based on the alignment of the H-cluster. ML bootstrap support values are indicated in the branches**. ML computation using a WAG+4 discrete-rate G+I+F model.

[FeFe]-hydrogenases are rather common in anaerobic Bacteria and Archaea, but in eukaryotes its presence is limited to a few species of anaerobic protists, anaerobic chytrid fungi and some green algae. In general, the hydrogenase is located in membrane-bounded organelles, i.e. plastids or hydrogenosomes. In *Giardia *and *Entamoeba *the enzyme is located in the cytoplasm [46]. [FeFe]-hydrogenases are generally monomeric and exhibit a multi-domain structure, with a very well conserved active site of ca. 350 residues - the H-cluster - and a variably sized N-terminal domain containing up to four Fe-S clusters [47]. This phylogeny was computed using the H-cluster portion of the protein, due to the modular structure of the hydrogenases, and it positions *Psalteriomonas lanterna *as a sister group of the algal and fungal hydrogenases (Fig.7).

### Pyruvate:Ferredoxin oxidoreductase (PFO)

**Figure 8:**
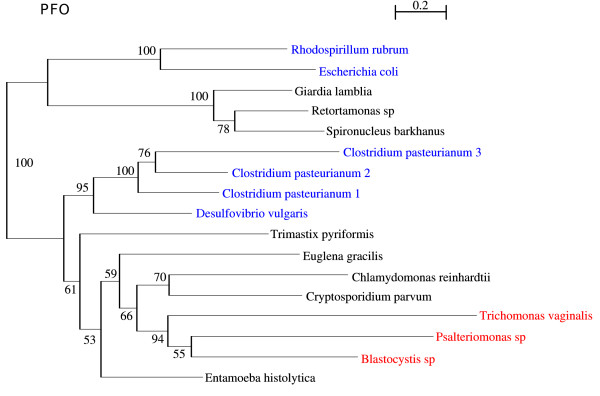
**Phylogenetic tree of the Pyruvate:ferredoxin oxidoreductase, computed by ML with WAG+4 discrete-rate G+I+F**. Branch support values represent bootstrap values.

Pyruvate is of central importance for the energy metabolism of cells. Its oxidative decarboxylation leads to the formation of acetyl-CoA and CO_2_. Aerobic species possess a pyruvate dehydrogenase (PDH) multi-enzyme complex, which catalyzes this reaction and specifically reduces NAD+. Anaerobic species in general use another set of specialized enzymes, which reduce low-potential electron carrier proteins, e.g. ferredoxin or flavodoxin, instead of pyridine nucleotides like NAD [48,49]. One of these enzymes is the pyruvate:ferredoxin oxidoreductase (PFO), which is present in many eubacteria and archaea, but also in a restricted number of anaerobic eukaryotes, like *Trichomonas vaginalis *[17], *Trimastix pyriformis *[28], *Giardia lamblia *and *Entamoeba histolytica *[50]. PFO seems not to be present in the aerobic amoeboflagellate *Naegleria gruberi*. It is regarded as a hallmark protein for hydrogenosomes or organisms with mitosomes [50], although PFO or the related PNO have also been detected in a few organisms with mitochondria, e.g. *Euglena *and *Chlamydomonas*. The PFO of *Psalteriomonas lanterna *branches with the enzymes of *Trichomonas vaginalis *and *Blastocystis*, but here is still some discussion regarding the intrinsic function of PFO [51] (Fig. 8). In *Blastocystis*, which belongs to the Straminopila and which possesses a hydrogenosome-like organelle, two EST clusters encoding a PFO and a PNO (pyruvate:NADP^+ ^oxidoreductase) were identified. Biochemical studies have so far provided only evidence for PNO activity [51].

### Propionyl-CoA carboxylase (PCCB)

Propionyl-CoA carboxylase is a biotin dependent enzyme that catalyses the ATP dependent carboxylation of propionyl-CoA to D-methylmalonyl-CoA. It is involved in the metabolism of odd-chained fatty-acids, cholesterol and the essential amino acids threonine, methionine, valine and isoleucine [52].

The PCC structure consists of two heterologous subunits, alpha and beta, encoded by PCCA and PCCB genes, respectively. The dodecamer enzyme complex is arranged in an alpha6beta6 conformation [53].

**Figure 9:**
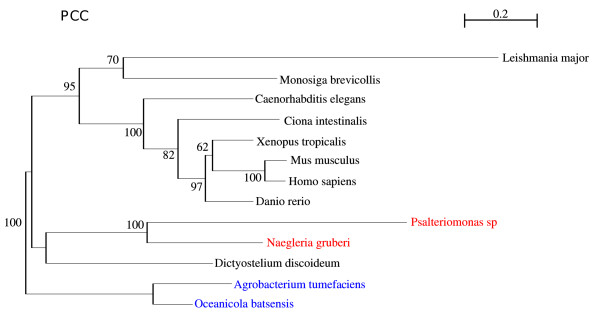
**ML phylogenetic tree of Propionyl Co-A carboxylase (PCCB)**. Branch values are bootstrap support. This tree is rooted by 2 archaeal species, and was computed using a WAG+4 discrete-rate G+I model.

PCC is involved in metazoan ubiquitous pathways; it has a patchy distribution among other Eukaryotes, suggesting multiple gene loss events [54,55] and it was included in our analysis, because of its location in the mitochondrial matrix and its pivotal metabolic role. The phylogenetic analysis shows a 100% bootstrap value for the clustering between *Psalteriomonas lanterna *and *Naegleria gruberi *(Fig. 9), which is consistent with the results obtained with Hsp60 and points to a close relationship between *Naegleria's *mitochondrion and *Psalteriomonas's *hydrogenosome.

### NADH: ubiquinone oxidoreductase 51 kDa subunit (Complex I -ndufv1/NuoF)

**Figure 10:**
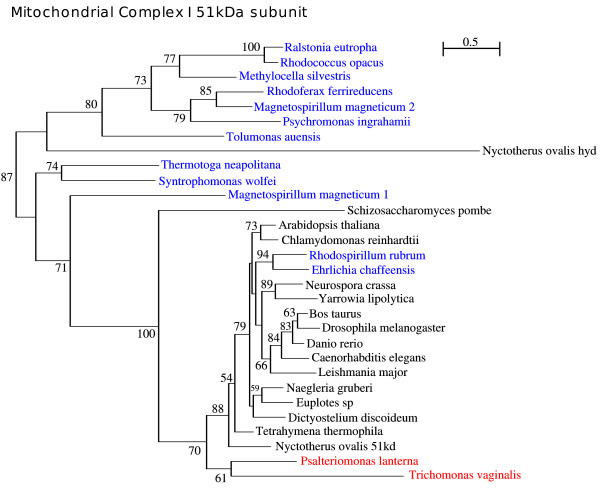
**NADH:ubiquinone oxidoreductase 51 kDa subunit ML phylogeny with bootstrap values indicated in the branches, and computed using a WAG+4 discrete-rate G**.

NADH: ubiquinone oxidoreductase, commonly known as mitochondrial Complex I, is the largest of the five OXPHOS complexes present in the mitochondrial membrane of aerobic organisms, comprising 45 proteins in human [56]. This protein complex can be divided into 3 functional modules: the dehydrogenase module which is responsible for the oxidation of NADH, the hydrogenase module that shuttles the released electrons, and finally, the transporter module, which pumps protons across the mitochondrial membrane [57]. The 51 kDa subunit, encoded by the *ndufv1/nuoF *nuclear gene, is an essential part of the dehydrogenase module because it carries the NADH binding site. Also it binds two co-factors: flavin mononucleotide (FMN) which is an electron carrier molecule that acts as a hydrogen acceptor and one 4Fe-4S cluster, which captures the electrons released from the NADH oxidation [58]. Despite its role in mitochondrial Complex I, this protein has been found in the absence of most of the remaining proteins of this complex in at least two organisms: *Trichomonas vaginalis *[59] and *Schizosaccharomyces pombe *[56], where it is believed to bind and oxidize NADH, potentially functioning as a diaphorase for the hydrogenase. Once again, the link between *Psalteriomonas *and *Trichomonas *is present in this phylogeny, showing also that the 51 kDa of *Psalteriomonas *belongs to the cluster of mitochondrial and alpha-proteobacterial enzymes (Fig. 10).

### Glutamate dehydrogenase (GDH)

**Figure 11:**
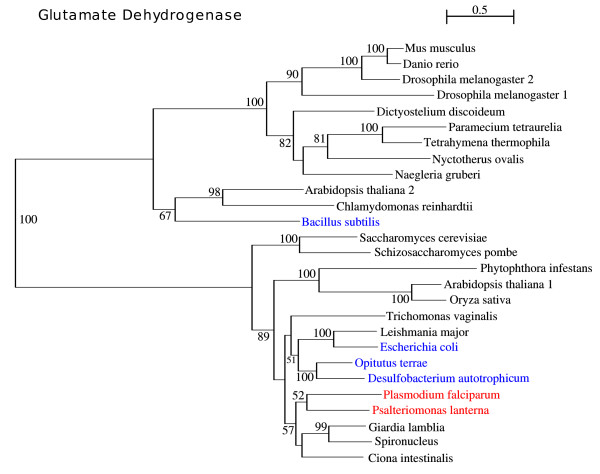
**Phylogenetic tree of the Glutamate dehydrogenase computed by ML with a RtREV+4 discrete-rate G+I+F**. Branches show the bootstrap support values.

Glutamate dehydrogenase (GDH) is a mitochondrial enzyme widely distributed in the three domains of life. It catalyzes the reversible oxidative deamination of glutamate to 2-oxoglutarate and ammonia, using either NAD or NADP as a co-factor. This enzyme is classified in three basic types, according to its co-factor specificity: the NAD specific, the NADP specific and the dual enzyme which accepts either of these. These enzymes are homopolymeres, commonly composed by two to six subunits [60]. While multicellular eukaryotes present only the dual enzyme, fungi have both co-factor specific enzymes and protists possess any combination of dual and specific GDHs [61]. The phylogenetic analysis clusters *Psalteriomonas *within the *Ciona*, *Spironucleus *and *Giardia *branch (Fig. 11). The position of *N. gruberi *relative to that of *Psalteriomonas *is at odds with their relation observed in the PCCB and the Hsp60 phylogeny. Glutamate dehydrogenases show evidence of frequent lateral gene transfer [61], providing an explanation for this inconsistency.

## Conclusion

The hydrogenosomes of *Psalteriomonas lanterna *are morphologically similar to the mitochondria of its relatives, the aerobic Heterolobosea, if one ignores the absence of cristae in *Psalteriomonas lanterna*. This becomes evident from their shape, the double membrane, which bounds both types of hydrogenosomes of *Psalteriomonas lanterna*, and the cisterns of rough ER that surround the cytoplasmic forms of the hydrogenosomes. This is characteristic for the mitochondria of the aerobic Heterolobosea, which, in contrast to the hydrogenosomes of *Psalteriomonas lanterna*, possess full-fledged cristae. However, there are no reports that the mitochondria of the aerobic Heterolobosea can form stacks like the hydrogenosomes of *P. lanterna*.

The molecular data, which are summarized in Table 1, also support the similarity between the hydrogenosomes of *P. lanterna *and the mitochondria of the aerobic Heterolobosea since the mitochondrial proteins Hsp60 and Propionyl-CoA Carboxylase B of *Psalteriomonas lanterna *are closely related to their homologues of *Naegleria gruberi*. The close phylogenetic relationship between *Psalteriomonas lanterna *and *Naegleria gruberi *had also been shown by the analysis of the 18S rRNA [21].

**Table 1:** Presence of hydrogenosomal genes of *P. lanterna *in various genomes

	Mitochondrion							Mitosome				Hydrogenosome			
	Hsa	Sce	Tth	Pte	Pfa	Ngr	Lma	Cpa	Ecu	Gla	Ehi	Tva	Nov	Pla	Nfr
EF1-alpha	Y	Y	Y	Y	Y	Y	Y	Y	Y	Y	Y	Y	Y	Y	Y
Hsp60	Y	Y	Y	Y	Y	Y	Y	Y	N	Y	Y	Y	Y	Y	Y
AAC	Y	Y	Y	Y	Y	Y	Y	Y	Y*	N	Y*	Y*	Y	Y*	Y
[FeFe]hydrogen.	N	N	N	N	N	Y	N	N	N	Y	Y	Y	Y	Y	Y
PFO	N	N	N	N	N	N	N	Y*	N	Y	Y	Y	N	Y	N
PCCB	Y	N	Y	Y	N	Y	Y	N	N	N	N	N	-	Y	-
51 kDa	Y	N	Y	Y	N	Y	Y	N	N	N	N	Y	Y	Y	-
GDH	Y	Y	Y	Y	Y	Y	Y	N	N	Y	Y	Y	Y	Y	-

Hsa: Homo sapiens, Sce: Saccharomyces cerevisiae, Tth: Tetrahymena thermophila, Pte: Paramecium tetraurelia, Pfa: Plasmodium falciparum, Ngr: Naegleria gruberi, Lma: Leishmania major, Cpa: Cryptosporidium parvum, Ecu: Encephalitozoon cuniculi, Gla: Giardia lamblia, Ehi: Entamoeba histolytica, Tva: Trichomonas vaginalis, Nov: Nyctotherus ovalis^$^; Pla: Psalteriomonas lanterna^$^, Nfr: Neocallimastix frontalis^$^.

Y: present, N: absent, Y*: alternative protein, -: not known;^$^: no complete genome available

**Figure 12:**
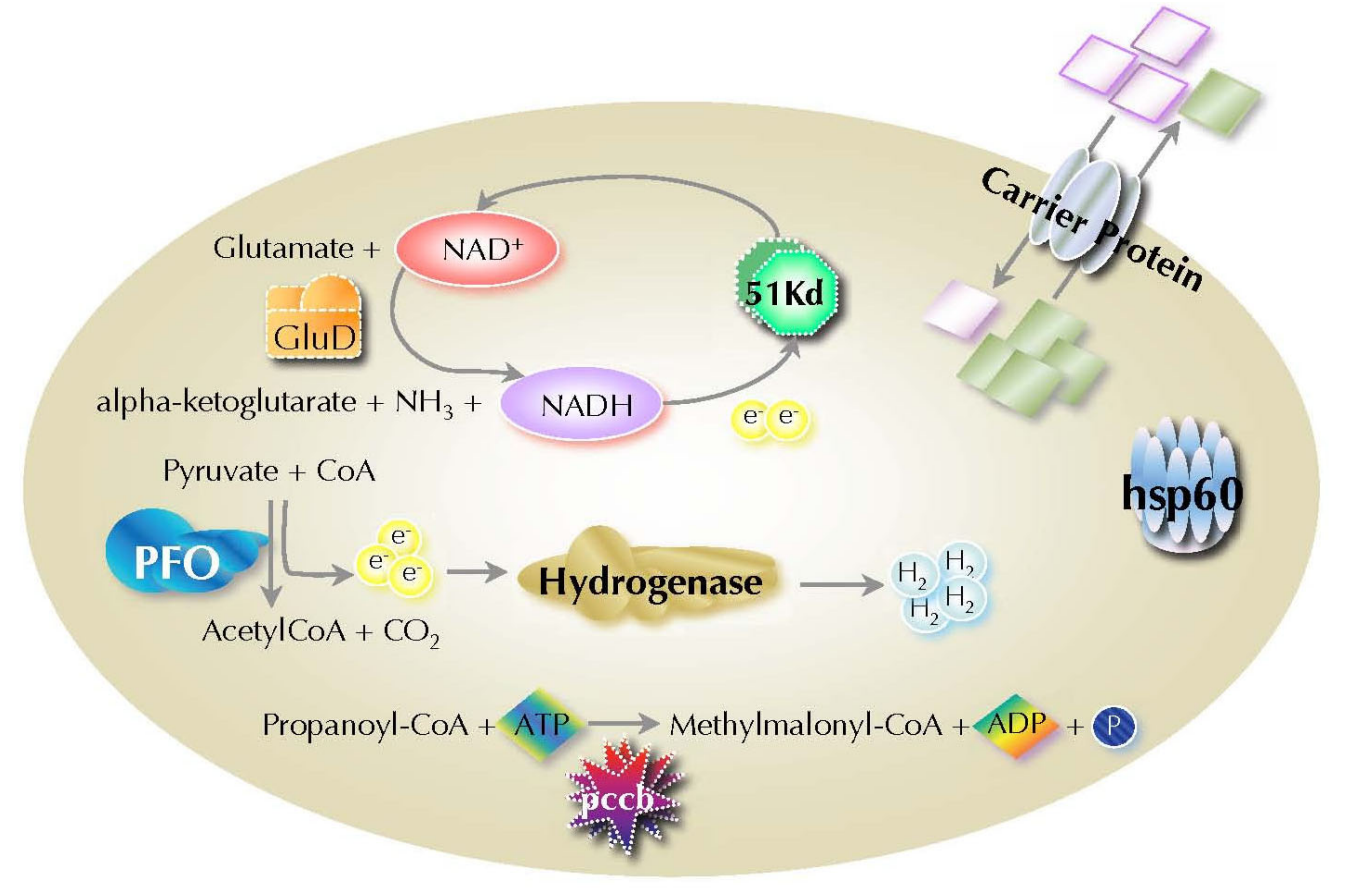
**Rudimentary metabolic scheme of the hydrogenosomes of *P. lanterna***.

The presence of a PFO, a [FeFe]hydrogenase, a putative alternative ADP/ATP translocator and the 51 kD subunit of mitochondrial complex I are characteristic hallmarks of a hydrogenosomal metabolism resembling that of *Trichomonas vaginalis *and *Trimastix pyriformis *[17,28]. However, this is the first report of a PFO in a hydrogenosome that is clearly derived from an aerobic mitochondrion. This allows the development of a rudimentary metabolic scheme (Fig. 12). The decarboxylation of pyruvate by PFO yields electrons, which, analogous to the situation in *Trichomonas *[17], requires a ferredoxin like protein similar to the one described earlier [27]. However, Blast analysis of the published ferredoxin sequence fails to reveal homologies with well-characterized ferredoxins. Moreover, the AT content of the gene is dramatically lower than that of other genes analysed in this study. Finally, an analysis of the codon usage with the aid of a principal component analysis clearly excludes the published ferredoxin sequence from the cluster of *Psalteriomonas *genes (not shown). Therefore, it is very unlikely that the published sequence is a *Psalteriomonas *ferredoxin, and, consequently, a genuine ferredoxin of *P. lanterna *still awaits detection.

Consequently, the transfer of the electrons from the PFO to the hydrogenase in our scheme (Fig. 12) remains unclear. It is possible that the 51 KDa protein might be involved since it can function as a diaphorase [17,59,62].

In conclusion, both the morphology of the hydrogenosomes and the molecular data strongly support the interpretation that the hydrogenosomes of *Psalteriomonas lanterna *and the mitochondria of the aerobic Heterolobosea share a common ancestor. The hydrogenosomes of *Trichomonas *are metabolically similar, but morphologically distinct, and they represent a peculiar type of hydrogenosome that lacks related mitochondrial relatives. Also the hydrogenosomes of ciliates and chytridiomycete fungi are different. And since the hydrogenosomes of the ciliate *Nyctotherus ovalis *share a common ancestry with ciliate mitochondria, while the hydrogenosomes of the anaerobic chytrids *Neocallimastix *and *Piromyces *share an ancestry with fungal mitochondria, our study provides a new example of the evolution of a hydrogenosome from an aerobic relative [14], and the first example of a *Trichomonas*-like hydrogenosome from an aerobic mitochondrion.

## Methods

### Cultivation of Psalteriomonas lanterna

*Psalteriomonas lanterna *was isolated from anoxic sediment from a sedimentation pond of a waste water treatment plant near Nijmegen about 20 years ago and cultured since then as a polyxenic culture. Bottles of 100 ml, 250 ml, 500 ml and 1000 ml were filled to 40% with 5 mM phosphate buffer (pH 6.8), 0.1 mM cysteine-HCl, 1 ml/l Pfennigs metal solution, 0.025% proteose pepton and Resazurine (50 pM w/v). The bottles were stoppered with butyl-rubber stoppers, evacuated, flushed with N_2 _and filled with this gas until a final pressure of 1.5 Bar. These bottles were sterilized and inoculated with *Psalteriomonas lanterna *cells. Oxygen was added until a final concentration of 1%. Twice a week bottles were checked; if they were completely anaerobic, oxygen was added up to 1%. The bottles were stored at 22°C and exposed to light every day for several hours.

### Generation of the EST library

Cells were harvested by centrifugation at room temperature in 50 ml glass tubes in a Hettich centrifuge for 5 minutes at 2000 r.p.m.. The supernatant was removed and the cell pellet was immediately dissolved in 8 M guanidiniumchloride (final concentration 6M). RNA was isolated and cleaned with the RNeasy Kit (Qiagen). The cDNA was created with the "SMART"- technology (BD Biosciences). The produced cDNA was amplified. Then the cDNA was restricted with Sfi I and size fractionated (fraction 1-2.5 kb and >2.5 kb). The DNA fragments were cloned site-directed. For the transformation we used competent *E. coli *DH10B cells. The library was generated by Genterprise, Mainz, Germany. About 480 randomly chosen clones were sequenced and analyzed using the BLAST X tool [36]. The A+T content was calculated, and clones with an A+T content of approximately 67-72% were regarded as derived from *Psalteriomonas lanterna*. The EST sequences varied largely in length and, in general, were incomplete.

### Generation of full-length cDNA

The sequence of the [FeFe] hydrogenase was nearly completed to the N- and C-terminal ends starting from the H-cluster (that was sequenced earlier [63]). Total RNA was isolated from *Psalteriomonas *cells using the RNeasy kit (Qiagen) according to the manufacturer's manual and subsequently, cDNA was generated using SuperScript (Invitrogen) and an anchored oligo-d(T) primer. Alternatively, SMART RNA amplification (Clontech) was used to generate (near) full-length cDNA sequences from all genes discussed here except 51 kDa.

### Electron microscopy

The electron microscopic preparations followed a modified Karnovsky procedure (4% paraformaldehyde and 5% glutardialdehyde in phosphate buffer pH 7.2). For postfixation, the OsO_4_/K_3_Fe(CN)_6 _method of Hepler [64] was applied. *En block *staining was performed with 2% uranyl acetate. After embedding in Epon 812 [65], sections were made on a Reichert Om U2 ultramicrotome, stained with lead citrate and uranyl acetate, and examined in a Zeiss 109 T electron microscope.

### Sequence data retrieval and alignment

The longest ORF from the conceptual translation (universal genetic code) of the ESTs of *Psalteriomonas *was obtained for each gene using Expasy's Translate tool http://www.expasy.org/tools/dna.html. The genes received the following GenBank accession numbers: 51kd: GQ924927, ADP/ATP carrier: GQ924928, PCCB: GQ924929, hydrogenase: GQ924930, PFO: GQ924931, elongation factor alpha: GQ924932, Hsp60: GQ924933, and Glutamate dehydrogenase GQ924934. Its homologous protein sequences were retrieved from GenBank nr database, using PsiBLAST [36] with 0.005 e-value cut-off and after three iterations. Sequences from *Naegleria gruberi *were collected from its genome project webpage http://genome.jgi-psf.org/Naegr1/Naegr1.home.html. Sequences were aligned with ClustalW (version 1.83) [66], and manually inspected and refined.

### Construction of phylogenetic trees

Given the large number of sequences retrieved by homology search, a restricted number of taxa, representing the major Eukaryotic branches, were selected to integrate the phylogenetic study. In order to facilitate this selection, a preliminary analysis of the complete dataset was carried out by inspecting the global topology of 1000 times bootstrapped Neighbour-Joining trees, computed with ClustalW [66]. When possible, a small outgroup of prokaryotic sequences was included to root the trees. After the careful selection of the final dataset, we pursued to select the best-fit model of amino acid replacement, according to the Akaike Information Criterion (AIC) as implemented in the ProtTest (version 2.2) software [67]. Maximum Likelihood (ML) phylogenies were computed with PhyML (version 2.4.4) [68], using the model previously chosen. (For the detailed description of the model and parameters used for each phylogenetic inference, including the matrix of aa substitution; number of Gamma discrete rate-categories (+G); proportion of invariable sites (+I) and observed amino acid frequencies (+F), see figure captions). A bootstrap analysis was conducted with 100 samples for each protein.

### Codon Usage and Principle Component Analysis

In order to rule out the presence of contaminants in the EST set, we performed a codon usage analysis using the Cusp program from the EMBOSS package (version 6.0.1) [37]. We also included in our analysis the ferredoxin sequence previously published by Brul et al. [27] to analyse whether it is of *Psalteriomonas *origin or not. A principle component analysis was conducted using the prcomp function of the R package (R Development Core Team, 2008).

## Authors' contributions

RMdG coordinated the molecular analysis, participated in sequencing of the ESTs and drafted the manuscript. ID analyzed the genetic code, performed the bioinformatic analysis and participated in drafting the manuscript. JR and JHPH performed the electron microscopy. TvA participated in sequencing and analyzing the ESTs and cultured the *Psalteriomonas *cells. JK and KS participated in cloning, sequencing and analyzing the hydrogenase gene. MAH supervised the phylogenetic analysis and participated in drafting the manuscript. JHPH initiated and coordinated the study and participated in drafting the manuscript. All authors read and approved the final manuscript.
